# Metabolomic changes in tear fluid following zinc biofortification in the BiZiFED nutritional study: a feasibility study

**DOI:** 10.3389/fmolb.2024.1421699

**Published:** 2024-09-10

**Authors:** Connor N. Brown, Babar Shahzad, Mukhtiar Zaman, Xiaobei Pan, Brian D. Green, Nicola M. Lowe, Imre Lengyel

**Affiliations:** ^1^ Wellcome-Wolfson Institute for Experimental Medicine, School of Medicine, Dentistry and Biomedical Sciences, Queen’s University Belfast, Belfast, United Kingdom; ^2^ Institute of Basic Medical Sciences, Khyber Medical University, Peshawar, Pakistan; ^3^ Department of Pulmonology, Rehman Medical Institute, Peshawar, Pakistan; ^4^ Institute for Global Food Security, School of Biological Sciences, Queen’s University Belfast, Belfast, United Kingdom; ^5^ Centre for Global Development, School of Sport and Health Sciences, University of Central Lancashire, Preston, United Kingdom

**Keywords:** zinc, nutrition, tear, LC-MS, BiZiFED, metabolomics

## Abstract

**Background:**

Biofortified Zinc Flour to Eliminate Deficiency in Pakistan (BiZiFED) is a nutritional research program that evaluates the impact of consuming zinc biofortified wheat flour on zinc status and associated health outcomes of vulnerable communities in northwest Pakistan. Measuring zinc status from blood samples is fraught with problems. This feasibility study evaluated whether metabolite changes in tear biofluids could be used to understand zinc status.

**Methods:**

Zinc deficiency is particularly prevalent amongst the female population in Pakistan. Therefore, a crossover trial was developed in which 25 women of reproductive age received standard, wheat flour, and another 25 received zinc-biofortified wheat flour for 8 weeks. At the end of this period, the nutritional intervention was switched between the groups for another 8 weeks. Tear biofluid was collected using Schirmer strips at baseline and after 8 and 16 weeks. Metabolomic analysis was conducted using the MxP^®^ Quant 500 kit on the tear biofluid from a subset of the study participants.

**Results:**

Two metabolites had a significantly negative correlation with plasma zinc concentration: tiglylcarnitine and valine. Compared to baseline metabolite concentrations, acetylcarnitine, glutamine, two lysophosphatidylcholines (lysoPC a C16:0 and lysoPC a C18:1), and four sphingomyelins (SM (OH) C16:1, SM C16:0, SM C16:1, and SM C24:0) were all significantly decreased post-zinc intervention, whilst a ceramide (Cer(d18:1/18:0) was significantly increased.

**Conclusion:**

These results highlight the potential of using tear biofluids as an alternative source for metabolomic biomarkers, both for the assessment of the zinc status of individuals enrolled in nutritional studies and for indicating physiological changes that arise from nutritional supplementation.

## Introduction

Micronutrient deficiencies affect approximately one-third of the global population (Pingault et al., 2017), with significant inequities in the burden of this ‘hidden hunger’, which particularly affects children and pregnant women in low- and middle-income countries (Wessells and Brown, 2012; Kassebaum et al., 2014; Micha et al., 2020). Amongst the micronutrients, zinc deficiency is well documented in Pakistan, with an average of 22.1% of women at reproductive age reported as being zinc deficient (Pakistan, 2019). Some of the well-documented negative impacts of zinc deficiency are slowed cognitive development, reduced immune competence, and complications during pregnancy and childbirth (Lowe, 2016), along with stunting of physical growth during development. These factors have been shown extensively in one marginalised rural community of Pakistan, the Peshawar community, where there is an above-average level of zinc deficiency in women of reproductive age (Brazier et al., 2020; Pakistan and UNICEF, 2020). The Peshawar community in Khyber Pakhtunkhwa have the highest proportion of children under 5 years of age with stunted growth (Pakistan and UNICEF, 2020), and there is also a correlation between the deficiency in plasma zinc concentration (PZC) and stunting in adolescent girls (aged 16–19 years old) of this community (Liere et al., 2017).

Strategies to improve zinc nutrition need to be developed, and work is ongoing to find solutions in the form of supplementation, dietary diversification, fortification, and biofortification. Of these options, fortification and biofortification appear to be the most likely strategies to gain traction as supplementation is often used as a therapeutic rather than a preventative strategy, and dietary diversification would require significant investment and programme development to support changes in food choices and behaviour that are limited by affordability (e-Pact Consortium, 2019; Gupta et al., 2020). Mass fortification involves the addition of micronutrients to food during processing. Fortification of flour with micronutrients (including zinc) at large commercial roller mills has been trialled in Pakistan, but success was limited because half of the population purchases their flour from local mills known as “Chakkis” rather than the large mills that are enrolled in the fortification initiative, thus severely limiting the reach of the program (e-Pact Consortium, 2021).

Biofortification of staple crops can be achieved through either transgenic techniques or conventional breeding to select varieties with naturally high micronutrient content, which can be combined with the addition of micronutrient fertilizer directly to the crop or soil to enhance the micronutrient content of the edible portion. Biofortification has the advantage that, once a high micronutrient variety has been developed, the farmer can retain a portion of the yield each year to use as seed for the following year. Thus, biofortification has the potential to have an impact on micronutrient deficiencies on a population scale. To date, six varieties of zinc biofortified wheat have been released onto the market in Pakistan. The first, known as Zincol-2016, was released in 2016 and was the variety evaluated in the Biofortified Zinc Flour to Eliminate Deficiency in Pakistan (BiZiFED) research program (Lowe et al., 2018, Lowe et al., 2020; Lowe et al., 2022), designed to explore the potential for zinc biofortified wheat to improve dietary zinc and iron intake for, and whether this correlates to functional alterations in the micronutrient status of women of reproductive age and adolescent girls (Lowe et al., 2020).

Zincol-2016 wheat grain, grown with the addition of zinc fertilizer to the soil and leaves, had a significantly elevated zinc concentration compared to standard (control) grain, resulting in a significant increase in the daily zinc intake from the flour compared with control flour, of between 3 and 6 mg per day for white and whole grain flour, respectively. However, it should be noted that this increase in zinc intake did not have a sustained impact on plasma zinc concentration or on fatty acid desaturase and elongase activity (FADS1 and 2), which has been explored as a putative biomarker of zinc status (Knez et al., 2017; Lowe et al., 2022).

Therefore, an alternative, sensitive biomarker is required to monitor better the physiological and biochemical impact of small changes in dietary zinc, such as those achievable through biofortification strategies to overcome these global health challenges. One such source of biomarkers is the tear fluid, which is increasingly being investigated as a non-invasive, cheap, physiologically relevant source of circulating biomarkers.

One of the first exclusive characterisations of the human tear metabolome employed a standard clinical method for tear collection coupled with an analytical platform to characterize the global repertoire of human tear metabolites (Chen et al., 2011). In this study, tears of healthy individuals were collected using the clinically utilized Schirmer strips, separated by ultra-fast liquid chromatography (LC), and analysed by quadrupole time-of-flight tandem mass spectrometry (Q-TOF MS/MS). This set a precedent for what could be achieved in tear metabolome studies, but it was clear that this method did not measure some well-known metabolites (e.g., measurement of glucose and ascorbic acid was affected by background interference).

Very few lipid species were identified, but within the literature, others have identified several classes of lipids using targeted analysis. These include free cholesterol (Borchman et al., 2007), phosphatidylcholines (Borchman et al., 2007; Saville et al., 2010), sphingomyelins (Borchman et al., 2007; Saville et al., 2010), wax esters (Borchman et al., 2007; Lam et al., 2013), lysophosphatidylcholine (Rantamäki et al., 2011; Dean and Glasgow, 2012), triacylglycerides, ceramides, and phosphatidylethanolamines (Dean and Glasgow, 2012). A further study investigated lipid composition during collection with Schirmer strips using untargeted analysis (Lam et al., 2014). Tears were collected by capillary tube or Schirmer strip, and extracted lipids were analysed using HPLC-MS. Over 600 lipid species across 17 lipid classes were detected, most categorized as wax or cholesteryl esters.

Schirmer strip collections yield the highest absolute amounts of lipids and are routinely employed in the clinic. Interestingly, the strips act as a chromatographic system for lipid metabolites (Rantamäki et al., 2011; Lam et al., 2014), whereby the aqueous fraction of the tear travels further along the strip than the non-polar lipids. The strips can accurately represent the lipidomic profile of tears and their relative concentrations when compared to spiking with artificial tear solutions (Lam et al., 2014).

As reviewed elsewhere (Khanna et al., 2022), tear samples appear to be a reasonable reservoir of metabolites. Their collection is non-invasive, and the samples are easy to handle and cheap to transport. These factors indicate that tear sampling could become a reliable source of metabolic markers for ophthalmic and systemic diseases (Cicalini et al., 2019; Valencia et al., 2022; Wen et al., 2023).

The aims of this study were threefold: first, to explore the feasibility of using tear biofluid as a source of metabolite biomarkers for human studies and the impact of transport and storage on sample viability; second, to assess whether metabolites in tear biofluid correlate with traditional zinc biomarkers; and thirdly, to assess the response of tear metabolites to small changes in dietary zinc intake across subgroups within a dietary zinc intervention program.

## Materials and methods

### Study location and design

**FIGURE 1:**
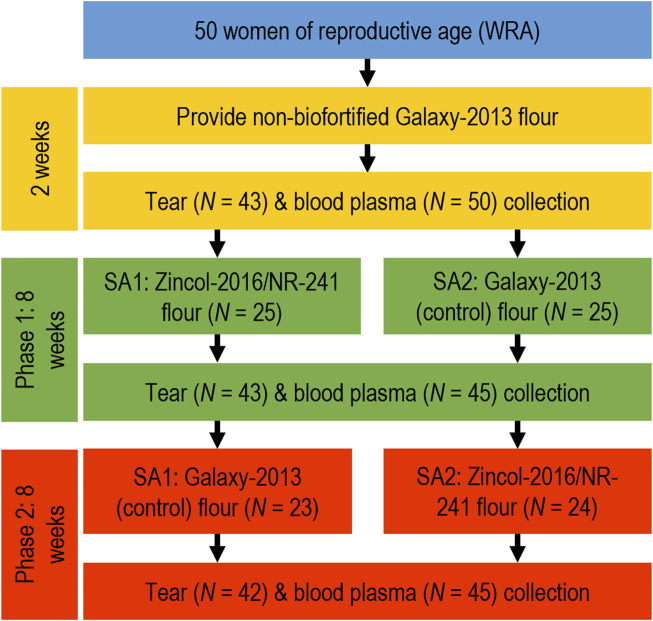
Schematic representation of the sampling schedule used to collect tear samples in a three-step process from families provided with a diet supplemented with flour derived from either Zincol-2016/NR-241 (intervention) grain or Galaxy-2013 (control) grain. SA–study arm.

This study was nested within the BiZiFED program, which included a double-blind, individually randomised, placebo-controlled study with cross-over design (Lowe et al., 2018; Ohly et al., 2019). The study was in a community close to the city of Peshawar in the province of Khyber Pakhtunkhwa in northwest Pakistan. This is a rural community where zinc deficiency is widespread and where the plant-based diet has a low zinc bioavailability. The trial was registered with the ISRCTN registry, study ID ISRCTN83678069 and undertaken between October 2017 and February 2018. The lead university (University of Central Lancashire; ethics reference no. STEMH 697 FR) and the collaborating institution in Pakistan, Khyber Medical University, granted ethical approval. The full study protocol has been previously described in detail (Lowe et al., 2022). In brief, 50 households, with at least one 16–49-year-old woman who was neither pregnant nor breastfeeding nor consuming additional nutritional supplementation, were randomly selected for the study. The selected households were provided with either freshly milled Zincol-2016/NR-421 grain (genetically and agronomically Zn-biofortified) or Galaxy-2013 grain (control), with the intention that these were the only flours to be consumed for the duration of the study. A 2-week baseline period was established with control flour before the households were randomly separated into two groups for the purposes of the study (Figure 1), one which received Zincol-2016/NR-421 grain for 8 weeks and then Galaxy-2013 control grain for 8 weeks (study arm 1 (SA1)), whilst the other received Galaxy-2013 control grain for 8 weeks followed by Zincol-2016/NR-421 grain (study arm 2 (SA2)) (Lowe et al., 2018).

### Participant information

At baseline, the characteristics of the study participants were recorded, including socio-economic status indicators and household demographics. In addition, dietary assessment was carried out using 24-h recalls throughout the 18-week study period using the multiple pass method, and detailed recipes for composite meals were collected for accurate curation of ingredients into an appropriate nutrient database. Thorough methodologies and results summarising demographic (Brazier et al., 2020) and dietary results (Lowe et al., 2022) have previously been published.

Where possible, blood was also collected from the study participants at the three time points, to determine plasma zinc concentrations pre- and post-dietary zinc intervention. Briefly, whole blood was collected into trace-element-free anticoagulant tubes and blood plasma was separated through centrifugation. Elemental concentrations of zinc were determined using inductively coupled plasma-mass spectrometry (ICP-MS) and reported recently (Brazier et al., 2020).

### Tear collection

Where possible, tears were collected on Schirmer tear test strips (4701001, Haag-Streit UK Ltd., Essex, UK) from both eyes of the study participants at all three time points. Briefly, the Schirmer strips were folded at the notch, before resting the rounded notch in the inferior conjunctival fornix (inside the lower eyelid) (Supplementary Figure S1A). Participants were given the option to close their eyes. The Schirmer strips were removed when the strips became saturated with tear fluid or once 5 min had passed, whichever came first. The strips were air dried, sealed in an envelope, placed in a Ziploc bag, shipped to Queen’s University Belfast (QUB) at ambient temperature, and stored at −80°C before analysis.

### Sample selection

To conduct a metabolomics feasibility and viability assessment from the tear samples obtained as part of the BiZiFED study, five samples were selected randomly from individuals who were initially enrolled on the study program but could not provide tear samples at all three time points. These were compared to freshly collected tear samples obtained from two in-house researchers simultaneously, with one sample collected from each eye from each person. Tears were collected in the same manner as those in the BiZiFED study. Once the tears were collected, the Schirmer strips were air-dried overnight at room temperature before metabolite extraction alongside the five randomly selected stored samples from the BiZiFED participants.

Of the remaining BiZiFED samples, a subset was selected for metabolomics analysis based on the following steps: 1) tear samples from individuals who could provide samples at all three time points were identified (*n* = 33); 2) tear samples from study participants were selected based on their plasma zinc status at baseline (*n* = 10). Plasma zinc concentration (PZC) is the most commonly used marker of zinc status (Abdulla, 1983) and a value of 660 μg/L was used as the threshold below which a participant was categorized as zinc-deficient (Brown et al., 2004). The methods and results to determine the PZC of BiZiFED study participants have previously been published (Brazier et al., 2020); 3) for LC-MS sample processing, a further 16 were randomly selected from the remaining 23 participants using a random number generator, to provide additional numbers inassessing whether tear metabolites responded to dietary zinc intervention in a larger cohort of individuals.

### Initial metabolite extraction and LC-MS procedure for assessing feasibility and viability of targeted metabolomics on stored tear fluid

To set up the protocol for metabolite extraction, two 4 mm punches were taken from each Schirmer strip (Supplementary Figure S1B), using a custom-made tool as per Dammeier *et al.* (Dammeier et al., 2018). Amino acids and acylcarnitines were extracted according to the manufacturer’s instructions using the MassChrom® kit (57000/F, ChromSystems Instruments & Chemicals, Munich, Germany). Once prepared for analysis, the material was reconstituted in 2.5 mM ammonium acetate in 25% methanol (aq) to analyse amino acids, after which the material was further dried down at 60°C and reconstituted in 2.5 mM ammonium acetate in 75% methanol (aq), to analyse lipids. Amino acids were injected through a MicroLC system coupled to a QTRAP 6500 mass spectrometer (AB Sciex, MA, United States), and lipids were analysed using flow injection analysis. Data analysis was carried out using the Morpheus web application (https://software.broadinstitute.org/morpheus/).

### Metabolite extraction and LC-MS procedure for targeted metabolomics

Based on the successful but variable results obtained from the punches, we deemed it necessary to extract metabolites from the Schirmer strip until the tear front was reached. As a first step, metabolites were extracted from tears collected at all three time points from one study participant. These were pooled together to assess whether the MxP® Quant 500 kit and the AB SCIEX Triple Quad 5500+ mass spectrometer were feasible methods for targeted metabolomics. In the following experiments, one Schirmer strip from each participant (*n* = 26) at the three time points (*n* = 78) was cut into 5 mm pieces until the tear front was reached and placed into sterile tubes. A length-adjusted volume of ice-cold 80% methanol was added to each tube (500 µL per 46 mm Schirmer strip). Strips were vigorously vortexed in the extraction solution for 2 min before centrifugation at 15,700 relative centrifugal force (RCF) at 4°C. The supernatant was transferred to a fresh, clean tube and stored at −80°C before analysis.

Targeted metabolomics profiling was performed using a commercially available kit, MxP® Quant 500 kit (Biocrates Life Science AG, Innsbruck, Austria), which quantifies up to 624 metabolites/lipids from 26 analyte classes. All frozen tear samples (−80°C) were thawed on ice before preparation. According to the instruction from the kit manufacturer, 10 µL of phosphate-buffered saline (PBS), calibrators, quality controls (QCs), and 10 µL of tear samples were added to a 96-well plate which contains isotopic-labelled internal standards, dried under nitrogen at room temperature for 30 mins, followed by adding 50 µL of phenylisothiocyanate (PITC) to derivatise amino acids and biogenic amines. After a further 20-min incubation at room temperature, samples were dried under nitrogen for 1 h. Ammonium acetate (3 mM) was added, and the plate was shaken at room temperature for 30 min and centrifuged for 2 min at 500 RCF. For LC-MS analysis, each well had 150 µL of extracts transferred to a 96-deep-well plate and an equal volume of dH_2_O. Metabolite separation was performed using an AB SCIEX ExionLC system (Foster, California, United States) with a reversed-phase MxP® Quant 500 UHPLC column and analysed using an AB SCIEX Triple Quad 5500+ mass spectrometer (Foster, California, United States) operating in the multiple reaction monitoring (MRM) mode. The injection volume is 5 µL. All the other metabolites (acylcarnitines, hexoses, glycerophospholipids, and sphingolipids) were quantified using the same mass spectrometer without column separation by the flow injection analysis (FIA) operating in MRM mode. A total of 10 µL of extract was mixed with 490 µL of FIA solvent in another 96-well plate at room temperature for 5 min. The injection volume is 20 µL. LC and FIA data were imported directly into the BIOCRATES software, MetIDQ Oxygen, and quantified for quantitation. Metabolite concentrations were calculated and expressed as micromoles (μM).

### Data analysis

**FIGURE 2:**
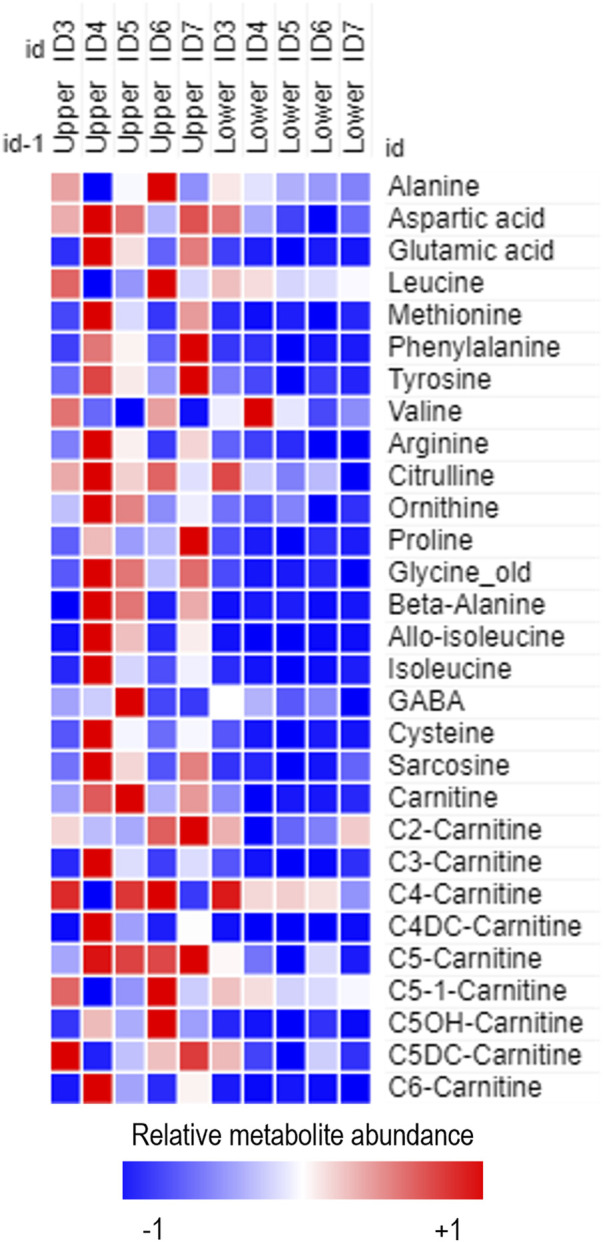
Heatmap of relative metabolite abundance in tear fluid obtained from the lower and upper portion of frozen Schirmer strip samples obtained from females of reproductive age enrolled on the BiZiFED study. Blue to red represents increasing metabolite abundance.

Data clean-up was performed to select metabolites above the detection limit accurately (>LOD) for further analysis. Metabolites that fell between the upper and lower limits of quantification (ULOQ and LLOQ) were also identified and were classed as valid metabolites (upplementary Figure 2). To understand whether there were any differences in the metabolites found in tear biofluid from the participants in the BiZiFED study, dependent on dietary Zincol-2016 supplementation, it was important to determine the PZC of the individuals included in the metabolomics analysis. Blood plasma zinc concentrations for each individual were determined by ICP-MS and reported elsewhere (Brazier et al., 2020).

Metabolite concentrations were calculated, and a linear mixed effects model was used to correlate each participant’s PZC at each time point to the paired tear metabolite concentrations. Here, we used time point (categorical) as a repeated measure and PZC (continuous) as a covariate of the metabolite concentration (continuous) as the dependent variable. Participant ID was applied as a random effect, and the β-coefficient prediction for the change in metabolite level per unit change in the levels of PZC was calculated using SPSS v29.0.0.0. Subsequently, to determine whether metabolite concentrations in tears responded to dietary zinc intervention, metabolite concentrations were normalised to the PZC of each participant at each time point and are expressed as micromoles per individual plasma zinc concentration (μM/[PZC]). Graphs of individual metabolites were plotted using GraphPad Prism software (v10.2.2). For analysis comparing metabolite concentrations pre- and post-zinc intervention, outliers were removed from each dataset following the ROUT method of outlier identification (Q = 1%). Statistical analysis of metabolite concentrations in Zn-deficient participants compared to Zn-efficient participants was done through one-way Brown-Forsythe and Welch ANOVA comparison with Dunnett’s T3 multiple comparisons testing. All other comparisons were through a pairwise *t*-test, or Wilcoxon signed rank test to determine significance, dependent on D’Agostino & Pearson normality testing.

## Results

### Participant information

As reported elsewhere (Brazier et al., 2020), the average age of the participating women was 35 ± 7 years, ranging between 22–48 years. Of the 47 participating women who completed the baseline data collection, all but one were illiterate, none were taking contraceptives, and only one woman had reported receiving medication in the month preceding the study. Additionally, more than half of the women were overweight or obese (66%), with a mean ± SD body mass index (BMI) at baseline of 27.1 ± 5.6 kg/m^2^. Further details of the dietary diversity of the participating individuals can be found in the corresponding research article (Brazier et al., 2020).

Of the 50 participants who were initially enrolled into the BiZiFED study, five participants withdrew from the study due to unwillingness to provide repeat blood samples (*n* = 2), migration out of the area (*n* = 1), or severe illness (*n* = 2). The remaining 45 participants were all able to provide blood samples at each time point (Lowe et al., 2022) (Figure 1). Seven participants declined to provide tear samples at baseline, seven participants declined to provide tear samples at phase 1, and eight participants declined to provide tear samples at phase 2 (Figure 1). Overall, 33 participants provided tear samples at all three time points. The reasons for declining tear collection at different stages was not recorded.

### Targeted metabolomics is feasible from tear samples subjected to long-term storage

The first preliminary experiment assessed whether targeted metabolomics was feasible on tear samples collected in the field and subjected to long-term storage conditions. Metabolites extracted from these samples were compared qualitatively to those extracted from freshly collected tear samples. Using the MassChrom® kit for targeted metabolomics, we showed that the abundance of metabolites was qualitatively variable on the upper portion of the strip between the freshly collected and stored tear samples (Supplementary Figure S3). This highlighted that storing tear samples on Schirmer strips provided viable metabolites upon extraction but should not be directly compared to freshly collected tear samples.

The second preliminary experiment assessed whether metabolite extractions should occur from the upper or lower portion of the Schirmer strip (Supplementary Figure S1B). From five female participants from the BiZiFED study, upper and lower punches were analysed using the MassChrom® kit for targeted metabolomics. Metabolites extracted and detected were more abundant in the upper portion of the strip (Figure 2), although, some metabolites had equal abundance on both the upper and lower portions of the Schirmer strips (including leucine, butyrylcarnitine (C4-carnitine) and 3-hydroxy-isovalerylcarnitine (C5OH-carnitine)) (Figure 2). This indicates that the Schirmer strip does not act as a chromatography system for all metabolites. We found intra-individual variabilities, as noted in the range of relative metabolite abundance found in the eyes of participant ID2 (Supplementary Figure S3A) and in participant ID1 (Supplementary Figure S3B). Variability in metabolite levels, such as we found in this study, has previously reported (Dammeier et al., 2018).

Based on the preliminary results, we determined that extracting metabolites from the entire Schirmer strip is necessary to provide as much metabolite coverage as possible in subsequent analysis. Next, we determined the number of metabolites we could detect in the BiZiFED samples. We used pooled tear samples (samples from the three time points) and analysed the metabolites present using the MxP® Quant 500 kit. This kit can quantify up to 624 metabolites, and we were able to identify 116 metabolites above the limit of detection (LOD) in the BiZiFED sample (Supplementary Figure S4A).

Next, we evaluated the number of analytes extracted from the 78 Schirmer strips we used from the BiZiFED study participant samples. We detected 67 metabolites after applying a strict validity cutoff (≥80% of samples above the metabolite LOD in any one analysis group (*n* ≥ 10)) (Supplementary Figure S4A). Overall, 11 of the 23 available metabolite classes had at least one valid metabolite for further analysis (Supplementary Figure S4B).

### Metabolites found in tear fluid correlate inversely with plasma zinc concentrations

**FIGURE 3:**
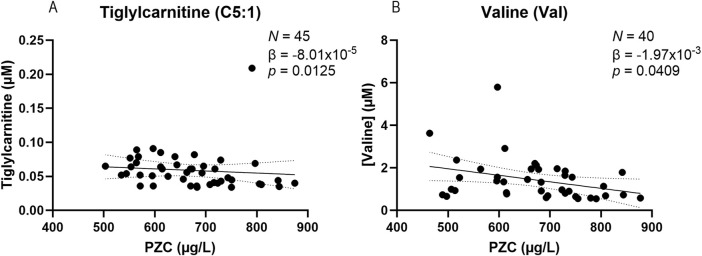
Scatter plots of tiglylcarnitine (C5:1) **(A)** and valine (Val) **(B)** obtained from tears of participants in the BiZiFED study which showed a significant negative correlation between metabolite abundance and plasma zinc concentration (PZC) (*N* = 40–45 tear samples, linear mixed effects model (β-coefficient)).

A linear mixed effects model was applied to determine whether there is a correlation between the concentration of metabolites found in tears and PZC (Figure 3). Two metabolites showed a weak but statistically significant correlation with PZC. Tiglylcarnitine (C5:1; *N* = 45) and valine (*N* = 40) were negatively correlated with PZC (β-coefficients = −8.01 × 10^−5^ and-1.97 × 10^−3^, respectively; *p* = 0.0125 and 0.0409, respectively) (Figures 3A, B, respectively). A summary of the correlations for the metabolites and PZC can be found in Supplementary Table S1, including 95% confidence intervals.

### Metabolites found in tear fluid respond to dietary zinc intervention

**FIGURE 4:**
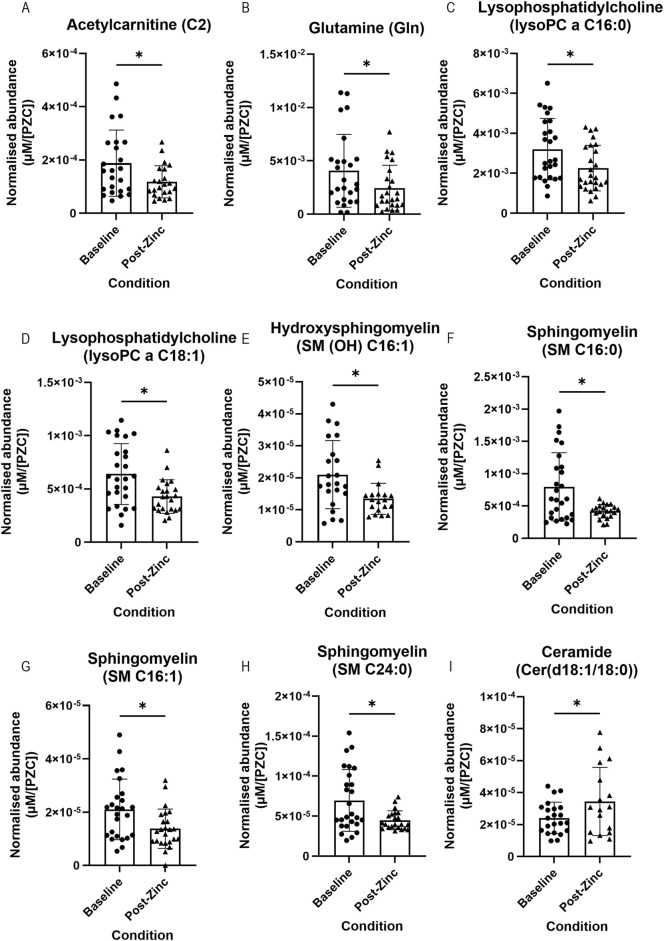
Representative bar graphs of metabolites collected from tears in the BiZiFED study that showed a significant difference in abundance between baseline (before zinc intervention) and post-zinc intervention. Metabolites are: acetylcarnitine (C2) **(A)**, glutamine (Gln) **(B)**, lysophosphatidylcholines (lysoPC a C16:0 and lysoPC a C18:1) **(C,D)**, respectively, sphingomyelins (SM (OH) C16:1, SM C16:0, SM C16:1, and SM C24:0) **(E–H)**, respectively, and a ceramide (Cer(d18:1/18:0)) **(I)** (*n* = 15–25 tear samples per experimental condition, mean ± S.D., *p < 0.05, pairwise *t*-test **(A–C,F–I)**, respectively or Wilcoxon signed rank test **(D,E)**, respectively.

We assessed whether dietary zinc intervention affected the metabolite abundances present in the tear fluid. Metabolite concentrations were normalised to each individual’s PZC and then to each individual’s respective baseline tear metabolite concentration. In total, nine metabolites showed a significant difference between baseline and post-zinc intervention (Figures 4A–I). Of these metabolites, acetylcarnitine (C2) (*n* = 20; 1.87 × 10^−4^ vs. 1.18 × 10^−4^ µM/[PZC]) glutamine (*n* = 23; 3.98 × 10^−3^ vs. 2.52 × 10^−3^ µM/[PZC]), two lysophosphatidylcholines (lysoPC a C16:0 (*n* = 25; 3.17 × 10^−3^ vs. 2.26 × 10^−3^ µM/[PZC]) and lyso a C18:1 (*n* = 23; 6.21 × 10^−4^ vs. 4.28 × 10^−4^ µM/[PZC])), and four sphingomyelins (SM (OH) C16:1 (*n* = 16; 1.98 × 10^−5^ vs. 1.36 × 10^−5^ µM/[PZC]), SM C16:0 (*n* = 21; 7.19 × 10^−4^ vs. 4.19 × 10^−4^ µM/[PZC]), SM C16:1 (*n* = 23; 1.98 × 10^−5^ vs. 1.40 × 10^−5^ µM/[PZC]), and SM C24:0 (*n* = 22; 6.71 × 10^−5^ vs. 4.48 × 10^−5^ µM/[PZC]) were all significantly downregulated (Figures 4A–H, respectively), and a ceramide (Cer(d18:1/18:0) was significantly upregulated (*n* = 15; 2.29 × 10^−5^ vs. 3.74 × 10^−5^ µM/[PZC]) (Figure 4I).

**FIGURE 5:**
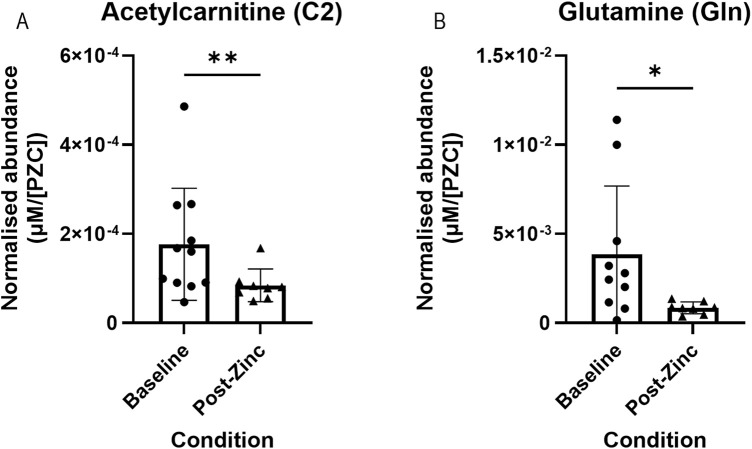
Representative bar graphs of metabolites collected from tears in the BiZiFED study that showed a significant difference in abundance between baseline and post-zinc supplementation in a zinc-deficient population. Metabolites are: acetylcarnitine (C2) **(A)** and glutamine (Gln) **(B)** (*n* = 7-9 tear samples per experimental condition, mean ± S.D., *p < 0.05, **p < 0.01, pairwise Wilcoxon signed rank test).

An additional comparison was made between metabolite abundances in the tears of all participants that were defined as having a low PZC (<660 μg/L) at their baselines (as per the iZINCG threshold (Brown et al., 2004)). Zinc intervention in this group resulted in significantly decreased concentrations of acetylcarnitine (C2) (*n* = 8; 1.92 × 10^−4^ vs. 8.45 × 10^−5^ µM/[PZC]) and glutamine (Gln) (*n* = 7; 2.43 × 10^−3^ vs. 8.58 × 10^−4^ µM/[PZC]) (Figures 5A, B, respectively).

#### Metabolites found in tear fluid respond to dietary zinc intervention in a subpopulation of zinc-deficient individuals

**FIGURE 6:**
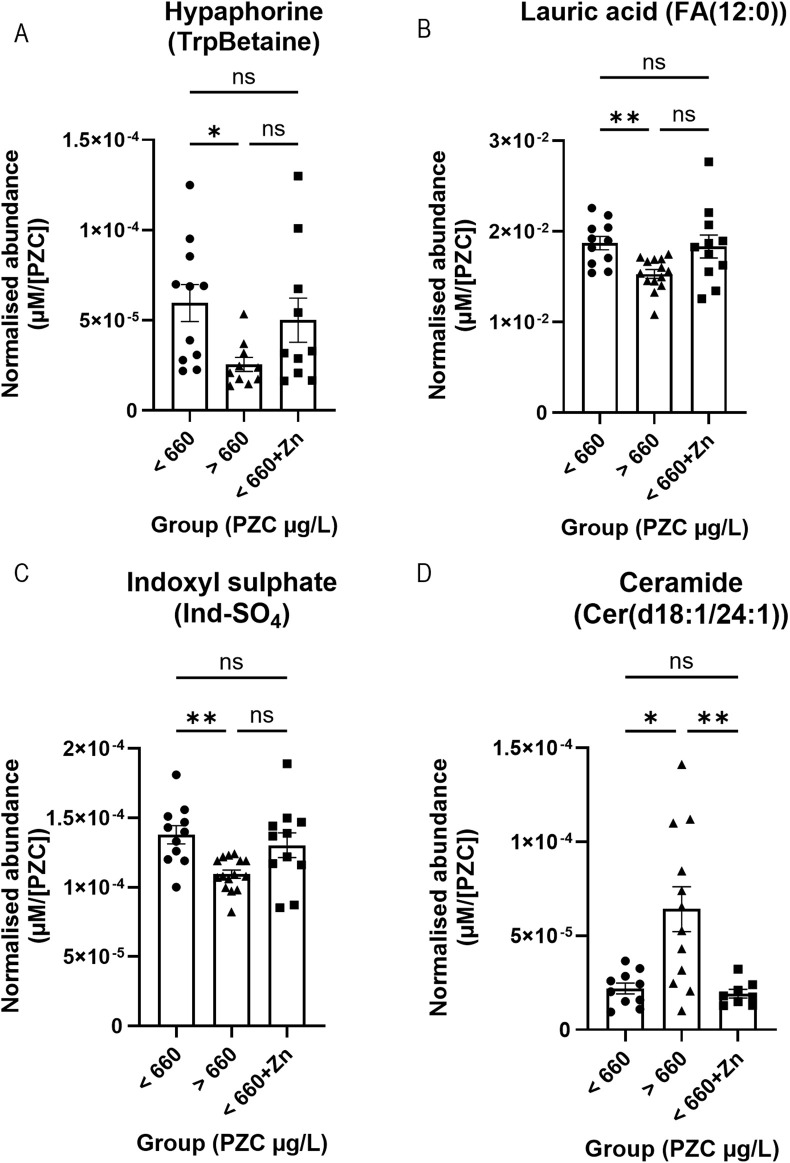
Representative bar graphs of metabolites collected from tears in the BiZiFED study that showed a significant difference in abundance between participants with PZC< 660 μg/L at baseline and post-zinc intervention, and those participants with PZC >660 μg/L at baseline. Metabolites are: Hypaphorine (TrpBetaine) **(A)**, lauric acid (FA (12:0)) **(B)**, indoxyl sulphate (Ind-SO_4_) **(C)**, and ceramide Cer(d18:1/24:1) **(D)** (*n* = 8–15 tear samples per experimental condition, mean ± S.D., *p < 0.05, **p < 0.01, ns = not significant, one-way ANOVA with Dunnett’s T3 multiple comparison testing).

The final set of comparisons were based on whether participants had PZC below or above the 660 μg/L iZINCG threshold for zinc deficiency. Four metabolites significantly differed at baseline (Figure 6). Hypaphorine (TrpBetaine) (*n* = 10–11; 5.96 × 10^−5^ vs. 2.55 × 10^−5^ µM/[PZC]), lauric acid (FA (12:0)) (*n* = 11–14; 1.87 × 10^−2^ vs. 1.53 × 10^−2^ µM/[PZC]), and indoxyl sulphate (Ind-SO_4_) (*n* = 11–15; 1.38 × 10^−4^ vs. 1.09 × 10^−4^ µM/[PZC])had significantly higher abundances (Figures 6A–C, respectively), whilst ceramide Cer(d18:1/24:1) was found at a significantly lower abundance (*n* = 10–12; 2.19 × 10^−5^ vs. 6.41 × 10^−5^ µM/[PZC]) (Figure 6D), in the tear fluid of zinc-deficient participants.

When dietary zinc intervention was provided to the zinc-deficient participants (<660+Zn), the concentrations of hypaphorine (*n* = 10; 5.00 × 10^−5^ vs. 2.55 × 10^−5^ µM/[PZC]) (Figure 6A), lauric acid (FA (12:0)) (*n* = 11–14; 1.83 × 10^−2^ vs. 1.53 × 10^−2^ µM/[PZC]) (Figure 6B), and indoxyl sulphate (Ind-SO_4_) (*n* = 11–15; 1.30 × 10^−4^ vs. 1.09 × 10^−4^ µM/[PZC]) (Figure 6C) were no longer different from those that were zinc-efficient at baseline (>660) but ceramide Cer(d18:1/24:1) remained different (Figure 6D). In addition, zinc supplementation values (<660+Zn) did not significantly differ from the metabolite level in zinc-deficient participants (<660) at baseline (Figures 6A–C). Individual mean concentrations for these metabolites can be found in Supplementary Table S2 (baseline) and Supplementary Table S3 (post-zinc intervention). Further studies exploring whether these changes remain in a larger cohort will be required before the biological mechanism and clinical relevance of these metabolite changes in response to zinc can be elucidated.

## Discussion

Zinc homeostasis is critical for the healthy function of many biological processes, including, but not limited to, growth, development, and neurological function or dysfunction. Importantly, zinc dysregulation has been shown to contribute to the development of progressive diseases such as age-related macular degeneration (AMD) and Alzheimer’s disease (AD), and global health issues arising from micronutrient deficiency (Maynard et al., 2005; Flinn et al., 2014; Lowe, 2021). Increasing dietary zinc intake to overcome deficiencies and associated pathophysiological consequences in these diseases continues to interest researchers (Age-Related Eye Disease Study Research Group, 2001; Brewer, 2014; Awh et al., 2015; Lowe et al., 2020), and sensitive biomarkers of zinc status are required. However, biomarker detection often relies on invasive blood withdrawal, which can be problematic to obtain, store, and transport in field studies. In addition, blood zinc concentrations are a poor indicator of zinc status.

Tear sampling is inexpensive and well-tolerated, and the resulting samples are easy to handle (Quah et al., 2014). Tears contain biomarkers that overlap with those detected in the systemic circulation (Ravishankar and Daily, 2022). Combined with recent improvements in separation and detection techniques (Cicalini et al., 2019; Valencia et al., 2022), it is unsurprising that tear fluid collection is now becoming a feature of study protocols for global health studies (Singh et al., 2022).

Our first aim in this study was to assess the feasibility of collecting, storing, and transporting tear samples, from a field-based nutrition study, which must be evaluated first by comparing them to freshly collected tear samples. We found that field-based samples contained a variety of metabolites (116 metabolites) despite being collected 1 year before extraction, and they were subjected to multiple freeze-thaw cycles because they had to be collected, transported, and stored at multiple locations (Lowe et al., 2018). When metabolites were extracted from a single Schirmer strip, the number of metabolites detected was lower (67 metabolites), suggesting that some low-abundance metabolites might not be reliably detected from a single strip, a consideration for future studies. While planning the metabolite extraction from the tears collected on Schirmer strips, it became apparent that current methods are not standardised, with each research article carrying out unique extraction and detection methods. This increases the difficulty of making inter-research group comparisons, as different extraction and detection methods will obtain varying metabolite classes and abundance of metabolites. The type of Schirmer strip used (García-Porta et al., 2018), sample handling (Qin et al., 2017), time to extraction, and extraction volumes (Gijs et al., 2023) can all influence detected biomarkers that have previously been obtained clinically. The Tear Research Network has recently been established to overcome these issues and develop clinically relevant standard operating procedures (https://tearresearchnetwork.com/).

Our second aim was to assess the relationship between plasma zinc concentrations and metabolite levels. Based on the results from 26 participants, our analysis revealed a significant negative correlation for two metabolites, tiglylcarnitine (C5:1) and valine, and PZC (Figure 3). Tiglylcarnitine is related to carnitine and is a member of the short-chain acylcarnitine group of metabolites. These have been suggested to be the most abundant group of acylcarnitines in human plasma (Dambrova et al., 2022) and carnitine levels in tears were associated with hyperosmolarity in dry eye diseases (Pescosolido et al., 2009). Elevated levels of tiglylcarnitine have been associated with liver zinc deficiency (Zheng et al., 2013). Valine concentrations have previously been shown to be increased in zinc-deficient rats (Hsu, 1977). It appears that our results agree with those inverse relationships between zinc and specific metabolites.

The third aim was to determine whether metabolites respond to dietary zinc intervention in the tear biofluid. First, we combined all selected participants (*n* = 26) before zinc intervention and compared them to those after zinc intervention. We found that the concentration of acetylcarnitine (C2), glutamine (Gln), two lysophosphatidylcholines (lysoPC a C16:0 and lysoPC a C18:1), an hydroxysphingomyelin (SM (OH) C16:1), and three sphingomyelins (SM C16:0, SM C16:1, and SM C24:0) significantly decreased, and a ceramide (Cer(d18:1/18:0)) significantly increased, post-zinc dietary intervention (Figure 4). Next, we analysed metabolic changes in only those zinc deficient at baseline (PZC <660 μg/L, *n* = 10). Again, we found that acetylcarnitine (C2) and glutamine had significantly decreased following zinc intervention in this subset of study participants (Figure 5). Comparison of tear samples from participants with PZC <660 μg/L (*n* = 10), zinc-sufficient participants with PZC >660 μg/L (*n* = 16), and those with PZC <660 μg/L, but after zinc supplementation, showed no reversal of zinc deficiency-related metabolic changes (Figure 6). This suggests that metabolic changes might need more time to restore to the levels found in zinc-sufficient participants.

The physiological consequences of the metabolic changes identified after zinc intervention are not yet clear. However, it is important to consider that using the PZC to measure zinc deficiency is fraught with problems as total zinc measurement may not be a sensitive enough parameter for patient selection (Lowe et al., 2022). The metabolites that were detected in our study and showed significant differences pre- and post-zinc dietary intervention have all previously been detected in tear metabolomic studies (Borchman et al., 2007; Nakatsukasa et al., 2011; Rantamäki et al., 2011; Dean and Glasgow, 2012; Glasgow and Abduragimov, 2018; Du and Huang, 2019), highlighting their potential use as biomarkers for zinc status.

Importantly, this study showed that it is feasible to use the non-invasive and inexpensive tear fluid collection to assess changes in zinc nutrition, which can generate valuable data for monitoring the effectiveness and impact of interventions designed to improve dietary intake. Some limitations should be considered. Firstly, this study used tear fluid obtained from a small cohort of individuals with additional limitations determined by the number of samples and metabolites that can be studied on the Biocrates MxP® 500 kit. It is also important to consider that using the PZC as a measure of zinc deficiency may not be a sensitive enough parameter for patient selection (Lowe et al., 2022). Using the data obtained from this study, a higher-powered study with a more targeted approach could be used to investigate metabolites that showed significant correlations with PZC or significant differences upon dietary zinc intervention.

In summary, we proved that it is feasible to carry out metabolite studies in field-based nutrition studies, using historically archived tear biofluid on Schirmer strips. After modified zinc nutrition there were significant differences in metabolites in both zinc sufficient and deficient patients. These might prove to biomarkers for zinc status in future studies. Given the wide-ranging role zinc plays in many biological processes (Costa et al., 2023; Shi et al., 2024), the availability of biomarkers could help refine intervention strategies. The diagnosis of zinc deficiency is challenging, and the search for a sensitive and specific biomarker has been ongoing for several decades. Further studies will help to define whether the metabolic changes reported here could be added to the list of zinc status biomarkers to increase sensitivity and specificity (Knez and Boy, 2023).

## Data Availability

The datasets presented in this study can be found in online repositories. The names of the repository/repositories and accession number(s) can be found below: http://dx.doi.org/10.21228/M8KT6Q.
