# Supplementary material for: Metabolomic changes in tear fluid following zinc biofortification in the BiZiFED nutritional study: a feasibility study
Source: Front Mol Biosci. 2024 Sep 10;11:1421699. doi: 10.3389/fmolb.2024.1421699 (PMC11420025; doi:10.3389/fmolb.2024.1421699)
Supplement: Supplementary file 1 [file DataSheet1.pdf]

Supplementary Material

Metabolomic changes in tear fluid following zinc biofortification in the BiZiFED nutritional study: a feasibility study.

Connor N. Brown[1], Babar Shahzad[2], Mukhtiar Zaman[3], Xiaobei Pan[4], Brian D Green[4], Nicola M. Lowe[5], Imre Lengyel[1*]

*** Correspondence:** Imre Lengyel: i.lengyel@qub.ac.uk

1.1 Supplementary Figures

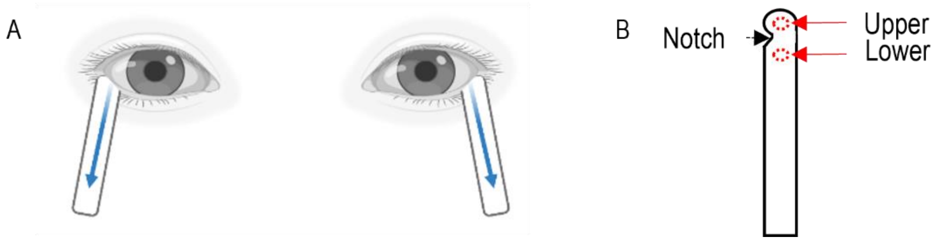

Supplementary Figure 1. The original arrangement of Schirmer strips, with the notched end placed between the lower eyelid and eye (A). Blue arrows indicate the direction of natural tear flow along the Schirmer strip. The shape of Schirmer strip and position of areas used for preliminary metabolite and protein identification (B).

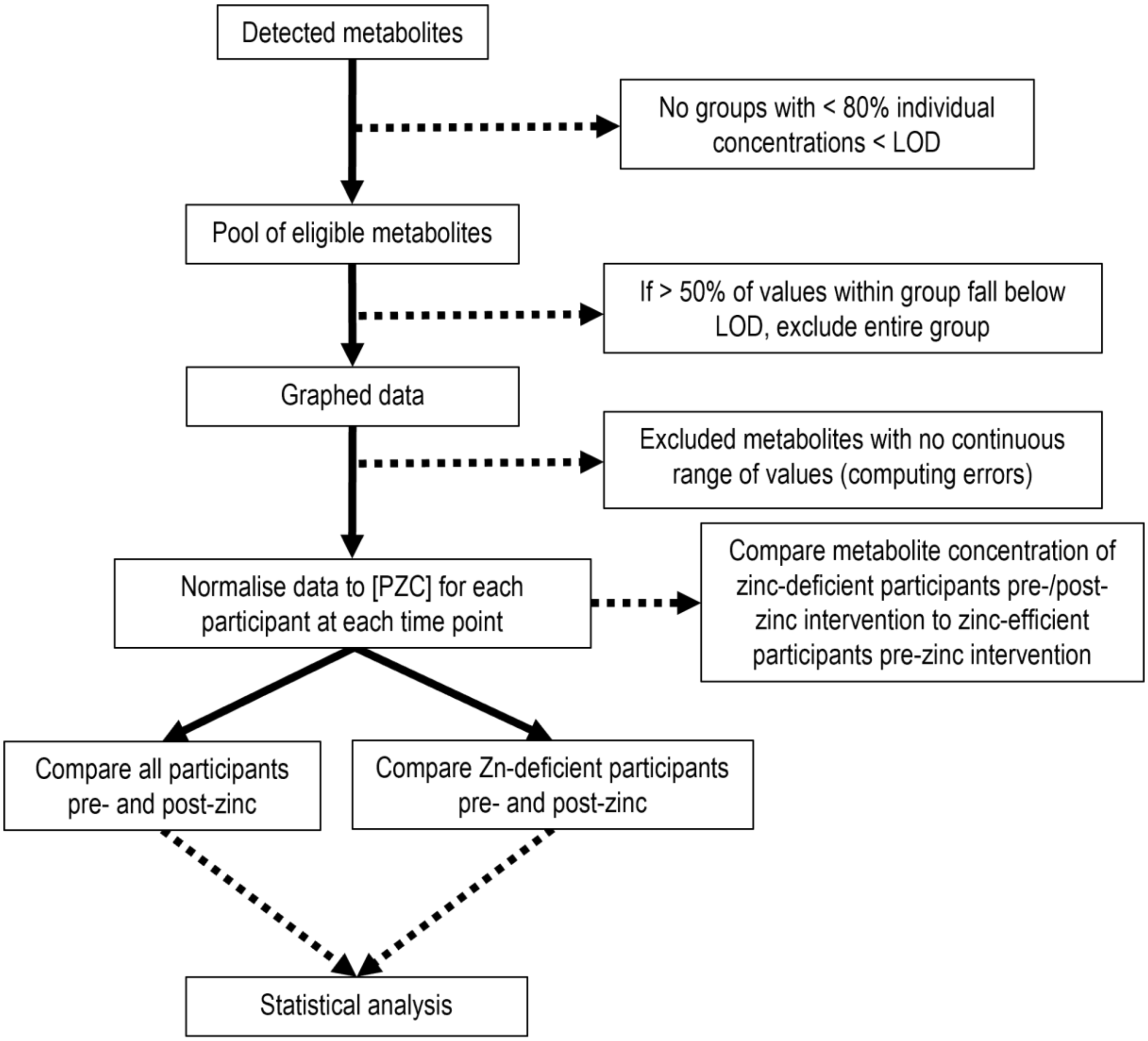

Supplementary Figure 2. Schematic representation of the data analysis workflow used to analyse the metabolites obtained from tears collected on Schirmer strips from participants in the BiZiFED study and processed collected using the Biocrates MxP® Quant 500 kit. LOD = limit of detection; PZC = plasma zinc concentration.

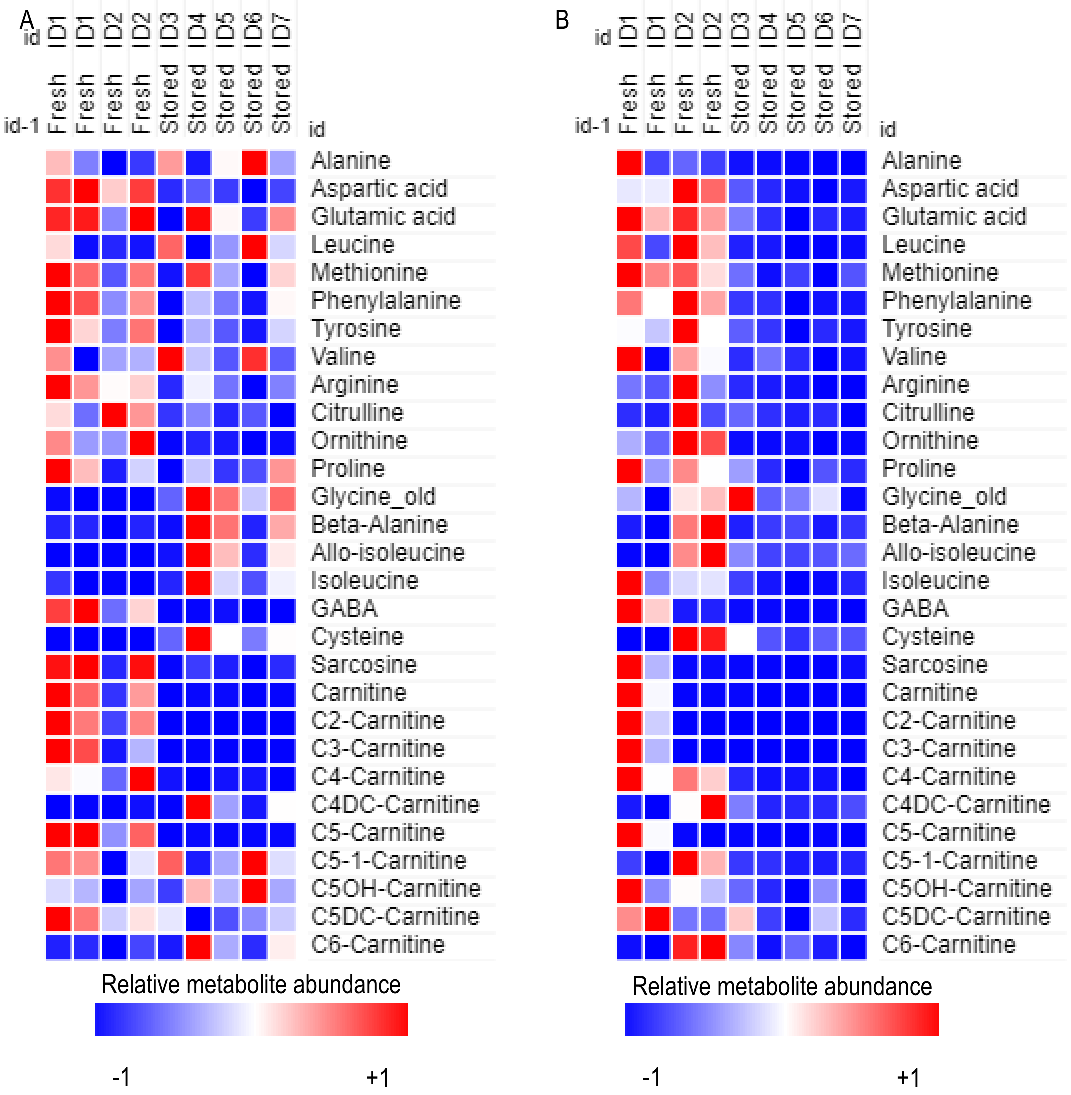

Supplementary Figure 3. Heatmap of relative metabolite abundance obtained from the upper (A) and lower (B) locations on Schirmer strips (see Supplementary Figure 1B). Tear samples were obtained from adult female participants enrolled in the BiZiFED study and fresh tear samples obtained on the same day as metabolite extraction. Blue to red represents increasing metabolite abundance.

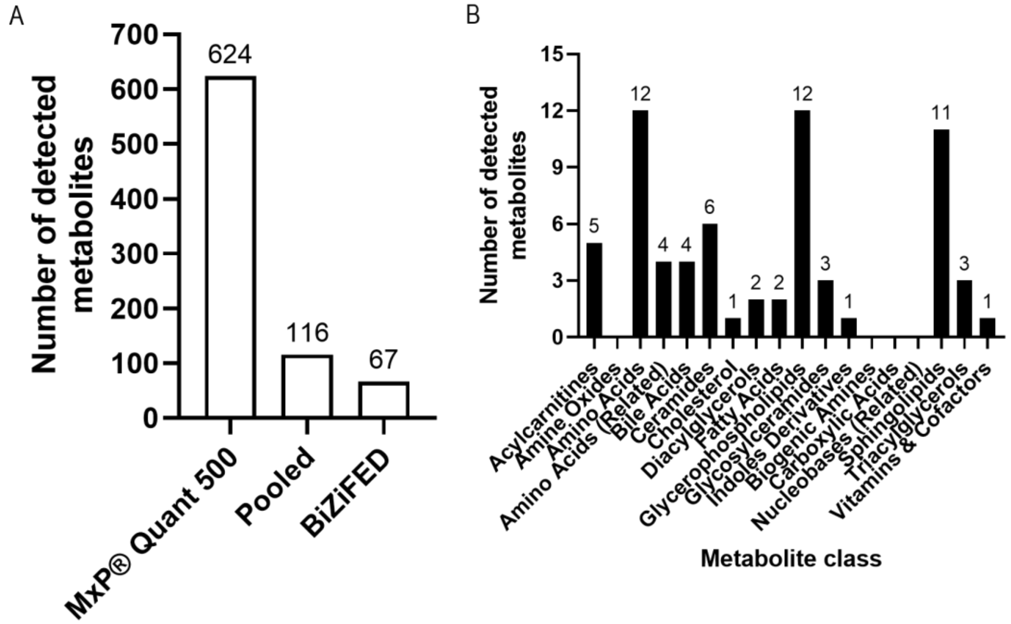

Supplementary Figure 4. Bar graphs representing the number of metabolites and metabolite classes detected using the Biocrates MxP® Quant 500 metabolomics analysis kit. A) Total number of metabolites detected by the MxP Quant 500 was compared to the number of metabolites detected in extracts from three pooled Schirmer strip samples or from the 78 individual Schirmer strip samples in the BiZiFED study (valid metabolites are those that fall between LLOQ and ULOQ in at least 80% of samples from one group). B) The number of metabolites detected in each compound class when extracted from the 78 Schirmer strip samples included in the BiZiFED study.

Supplementary Table 1. List of metabolites that were found to significantly correlate with plasma zinc concentration (PZC) using a linear mixed effects model with subject ID as a random effect.

PZC vs Metabolite abundance	Time point			Prediction	95% CI	*p* -value
	2 weeks	10 weeks	18 weeks			
PZC (mean µg/L (SD))	717.8 (58.5)	706.6 (100.7)	617.9 (79.13)	N/A	N/A	N/A
Tiglylcarnitine (mean µM (SD))	0.0529 (0.0133)	0.0492 (0.0195)	0.0689 (0.0361)	$-8.07x10^{-5}$	$-1.43x10^{-4}$ to $-1.83x10^{-5}$	0.012492
PZC (mean µg/L (SD))	689.6 (110.7)	681.3 (110.1)	614.6 (87.63)	N/A	N/A	N/A
Valine	1.13 (0.534)	1.44 (0.812)	1.93 (1.63)	$-1.97x10^{-3}$	$-3.86x10^{-3}$ to $-8.61x10^{-5}$	0.040871

Supplementary Table 2. List of metabolites found in tear fluid that are significantly altered between participants of the BiZiFED study with "low" and "normal" PZC at baseline.

Metabolite	Group	Mean metabolite concentration (μM/[PZC])	Standard deviation (± S.D.)	*p* -value
Hypaphorine (TrpBetaine)	PZC ≤ 660 μg/L	$5.96x10^{-5}$	$3.40x10^{-5}$	0.02
	PZC ≥ 660 μg/L	$2.55x10^{-5}$	$1.23x10^{-5}$	
Taurodeoxycholic acid (TCDA)	PZC ≤ 660 μg/L	$1.68x10^{-6}$	$9.92x10^{-8}$	<0.001
	PZC ≥ 660 μg/L	$1.36x10^{-6}$	$1.08x10^{-7}$	
Lauric acid (FA(12:0))	PZC ≤ 660 μg/L	$1.87x10^{-2}$	$2.43x10^{-3}$	0.002
	PZC ≥ 660 μg/L	$1.53x10^{-2}$	$1.82x10^{-3}$	
Indoxyl sulphate (Ind-SO_4)	PZC ≤ 660 μg/L	$1.38x10^{-4}$	$2.18x10^{-5}$	0.003
	PZC ≥ 660 μg/L	$1.09x10^{-4}$	$1.18x10^{-5}$	
Ceramide d18:1/24:1 (Cer(d18:1/24:1))	PZC ≤ 660 μg/L	$2.19x10^{-5}$	$9.19x10^{-6}$	0.01
	PZC ≥ 660 μg/L	$6.41x10^{-5}$	$4.14x10^{-5}$	

Supplementary Table 3. List of metabolites found in tear fluid that are not significantly altered between participants of the BiZiFED study when "low" PZC participants receive dietary zinc intervention and are compared to "normal" PZC participants at baseline.

Metabolite	Group	Mean metabolite concentration (μM/[PZC])	Standard deviation (± S.D.)	*p* -value
Hypaphorine (TrpBetaine)	PZC ≤ 660 μg/L	$5.00x10^{-5}$	$3.87x10^{-5}$	0.15
	PZC ≥ 660 μg/L	$2.55x10^{-5}$	$1.23x10^{-5}$	
Lauric acid (FA(12:0))	PZC ≤ 660 μg/L	$1.83x10^{-2}$	$4.21x10^{-3}$	0.08
	PZC ≥ 660 μg/L	$1.53x10^{-2}$	$1.82x10^{-3}$	
Indoxyl sulphate (Ind-SO_4)	PZC ≤ 660 μg/L	$1.30x10^{-4}$	$2.96x10^{-5}$	0.09
	PZC ≥ 660 μg/L	$1.09x10^{-4}$	$1.18x10^{-5}$	
